# Supplementary material for: Hemifusomes and interacting proteolipid nanodroplets mediate multi-vesicular body formation
Source: Nat Commun. 2025 May 17;16:4609. doi: 10.1038/s41467-025-59887-9 (PMC12085569; doi:10.1038/s41467-025-59887-9)
Supplement: Supplementary file 2 — Description of Additional Supplementary Files [file 41467_2025_59887_MOESM2_ESM.pdf]

Description of Additional Supplementary Files:

Supplementary Movie 1: Tomographic reconstruction of direct hemifusome in a COS-7 cell. Direct hemifusome in a COS-7 cell reveal a dense structure embedded within the hydrophobic interior of the bilayers at the junction of HD and two heterotypic vesicles. Video is displayed at 24 fps. Scale bar: 100 nm.

Supplementary Movie 2: Tomographic reconstruction of direct hemifusome in a COS-7 cell. Direct hemifusome in a COS-7 cell reveal a dense structure embedded within the hydrophobic interior of the bilayers at the junction of HD and two heterotypic vesicles. Video is displayed at 24 fps. Scale bar: 100 nm.

Supplementary Movie 3: Tomographic reconstruction of flipped hemifusome in a COS-7 cell. Flipped hemifusome in a COS-7 cell show a translucent vesicle hemifused to the luminal or exoplasmic side of the larger vesicle membrane. The videos are displayed at 24 fps. Scale bar: 100 nm.

Supplementary Movie 4: Time-lapse phase-contrast microscopy of the COS-7 cell edge. This time-lapse (6-minutes total recording) phase-contrast light microscopy movie) captures the dynamic edge of a COS-7 cell. The video highlights numerous birefringent and subdiffraction-limited vesicles, which are predominantly detectable only while in motion. The movie is representative of the regions sampled using cryo-ET and showcases the high dynamicity of the endomembrane system within this cellular region were hemifusomes were observed. As estimated from the cryo-ET data hemifusomes correspond to ~10% of the smaller vesicles in the region. Scale bar: 1μm.
