# Supplementary material for: Hemifusomes and interacting proteolipid nanodroplets mediate multi-vesicular body formation
Source: Nat Commun. 2025 May 17;16:4609. doi: 10.1038/s41467-025-59887-9 (PMC12085569; doi:10.1038/s41467-025-59887-9)
Supplement: Supplementary file 7 — Reporting Summary [file 41467_2025_59887_MOESM7_ESM.pdf]

nature portfolio

Corresponding author(s): Bechara Kachar

Last updated by author(s): Mar 11, 2025

Reporting Summary

Nature Portfolio wishes to improve the reproducibility of the work that we publish. This form provides structure for consistency and transparency in reporting. For further information on Nature Portfolio policies, see our Editorial Policies and the Editorial Policy Checklist.

Statistics

For all statistical analyses, confirm that the following items are present in the figure legend, table legend, main text, or Methods section.

n/a	Confirmed	
☐	☒	The exact sample size (*n*) for each experimental group/condition, given as a discrete number and unit of measurement
☐	☒	A statement on whether measurements were taken from distinct samples or whether the same sample was measured repeatedly
☒	☐	The statistical test(s) used AND whether they are one- or two-sided<br>*Only common tests should be described solely by name; describe more complex techniques in the Methods section.*
☒	☐	A description of all covariates tested
☒	☐	A description of any assumptions or corrections, such as tests of normality and adjustment for multiple comparisons
☐	☒	A full description of the statistical parameters including central tendency (e.g. means) or other basic estimates (e.g. regression coefficient) AND variation (e.g. standard deviation) or associated estimates of uncertainty (e.g. confidence intervals)
☒	☐	For null hypothesis testing, the test statistic (e.g. F, t, r) with confidence intervals, effect sizes, degrees of freedom and P value noted<br>*Give P values as exact values whenever suitable.*
☒	☐	For Bayesian analysis, information on the choice of priors and Markov chain Monte Carlo settings
☒	☐	For hierarchical and complex designs, identification of the appropriate level for tests and full reporting of outcomes
☒	☐	Estimates of effect sizes (e.g. Cohen's *d*, Pearson's *r*), indicating how they were calculated

Our web collection on statistics for biologists contains articles on many of the points above.

Software and code

Policy information about availability of computer code

Data collection	Tomography/5.6.0.2368REL, DigitalMicrograph/3.32.2403.0
Data analysis	GraphPad Prism/10, IMOD/4.12.25, ImageJ/1.54c, Amira/2024.1, ChimeraX/1.6

For manuscripts utilizing custom algorithms or software that are central to the research but not yet described in published literature, software must be made available to editors and reviewers. We strongly encourage code deposition in a community repository (e.g. GitHub). See the Nature Portfolio guidelines for submitting code & software for further information.

Data

Policy information about availability of data

All manuscripts must include a data availability statement. This statement should provide the following information, where applicable:

- Accession codes, unique identifiers, or web links for publicly available datasets
- A description of any restrictions on data availability
- For clinical datasets or third party data, please ensure that the statement adheres to our policy

The tomograms displayed in Movies 1–3 have been deposited in the Electron Microscopy Data Bank (EMDB) under accession numbers EMD-47672, EMD-47671, and EMD-47670, respectively. All other data required to evaluate the conclusions of this study are included within the paper. Cryo-EM data will be submitted to EMPIAR upon publication.

Research involving human participants, their data, or biological material

Policy information about studies with human participants or human data. See also policy information about sex, gender (identity/presentation), and sexual orientation and race, ethnicity and racism.

Reporting on sex and gender	NA
Reporting on race, ethnicity, or other socially relevant groupings	NA
Population characteristics	NA
Recruitment	NA
Ethics oversight	NA

Note that full information on the approval of the study protocol must also be provided in the manuscript.

Field-specific reporting

Please select the one below that is the best fit for your research. If you are not sure, read the appropriate sections before making your selection.

- [x] Life sciences
- [ ] Behavioural & social sciences
- [ ] Ecological, evolutionary & environmental sciences

For a reference copy of the document with all sections, see nature.com/documents/nr-reporting-summary-flat.pdf

Life sciences study design

All studies must disclose on these points even when the disclosure is negative.

Sample size	In 308 tomograms, we were able to identify clearly 88 direct hemifusomes and 48 hemifusomes in the flipped configuration. We also observed (at least 10 examples in 308 tomograms) other vesicles and lipid droplets, either docked (or hemifused).
Data exclusions	For cryo-EM, all micrographs were checked manually after motion correction , micrographs affected by drift or blockage were excluded from reconstruction.
Replication	Multiple cryo-EM data sets with freshly prepared and frozen samples were collected on about forty 24-hr Krios sessions using a semiautomatic data-acquisition strategy.
Randomization	For the cryo-ET and cryo-EM, the targeting positions for data collection were choose manually on grids for data quality control.
Blinding	Blinding was not used for the cryo-ET and cryo-EM studies, the investigators were aware of the identity of the samples

Reporting for specific materials, systems and methods

We require information from authors about some types of materials, experimental systems and methods used in many studies. Here, indicate whether each material, system or method listed is relevant to your study. If you are not sure if a list item applies to your research, read the appropriate section before selecting a response.

Materials & experimental systems

n/a	Involved in the study	
x		Antibodies
	x	Eukaryotic cell lines
x		Palaeontology and archaeology
x		Animals and other organisms
x		Clinical data
x		Dual use research of concern
x		Plants

Methods

n/a	Involved in the study	
x		ChIP-seq
x		Flow cytometry
x		MRI-based neuroimaging

Eukaryotic cell lines

Policy information about cell lines and Sex and Gender in Research

Cell line source(s)	COS-7 (ATCC CRL-1651), HeLa (ATCC CCL-2), NIH/3T3 cells (ATCC CRL-1658) were obtained from ATCC. Rat1 cells stably expressing claudin2-GFP were a gift from Dr. James Anderson's lab, which created the stable expressing cells using Rat1 obtained from ATCC.
Authentication	The cell lines obtained form ATCC were certificated by ATCC.
Mycoplasma contamination	All four cell lines tested negative for mycoplasma contamination using the mycoplasma PCR detection kit (abm, G238).
Commonly misidentified lines (See ICLAC register)	N/A

Plants

Seed stocks	NA
Novel plant genotypes	NA
Authentication	NA
